# Findings of the Chronic Obstructive Pulmonary Disease-Sitting and Exacerbations Trial (COPD-SEAT) in Reducing Sedentary Time Using Wearable and Mobile Technologies With Educational Support: Randomized Controlled Feasibility Trial

**DOI:** 10.2196/mhealth.9398

**Published:** 2018-04-11

**Authors:** Mark W Orme, Amie E Weedon, Paula M Saukko, Dale W Esliger, Mike D Morgan, Michael C Steiner, John W Downey, Lauren B Sherar, Sally J Singh

**Affiliations:** ^1^ Centre for Exercise and Rehabilitation Science National Institute for Health Research Leicester Biomedical Research Centre - Respiratory Leicester United Kingdom; ^2^ National Centre for Sport and Exercise Medicine Loughborough United Kingdom; ^3^ School of Sport, Exercise and Health Sciences Loughborough University Loughborough United Kingdom; ^4^ Department of Social Sciences School of Social, Political and Geographical Sciences Loughborough University Loughborough United Kingdom; ^5^ National Institute for Health Research Leicester Biomedical Research Centre Leicester United Kingdom

**Keywords:** chronic obstructive pulmonary disease, feasibility, fitness trackers, intervention, physical activity, sedentary lifestyle, sedentary time, self-monitoring, wearable electronic devices

## Abstract

**Background:**

Targeting sedentary time post exacerbation may be more relevant than targeting structured exercise for individuals with chronic obstructive pulmonary disease. Focusing interventions on sitting less and moving more after an exacerbation may act as a stepping stone to increase uptake to pulmonary rehabilitation.

**Objective:**

The aim of this paper was to conduct a randomized trial examining trial feasibility and the acceptability of an education and self-monitoring intervention using wearable technology to reduce sedentary behavior for individuals with chronic obstructive pulmonary disease admitted to hospital for an acute exacerbation.

**Methods:**

Participants were recruited and randomized in hospital into 3 groups, with the intervention lasting 2 weeks post discharge. The Education group received verbal and written information about reducing their time in sedentary behavior, sitting face-to-face with a study researcher. The Education+Feedback group received the same education component along with real-time feedback on their sitting time, stand-ups, and steps at home through a waist-worn inclinometer linked to an app. Patients were shown how to use the technology by the same study researcher. The inclinometer also provided vibration prompts to encourage movement at patient-defined intervals of time. Patients and health care professionals involved in chronic obstructive pulmonary disease exacerbation care were interviewed to investigate trial feasibility and acceptability of trial design and methods. Main quantitative outcomes of trial feasibility were eligibility, uptake, and retention, and for acceptability, were behavioral responses to the vibration prompts.

**Results:**

In total, 111 patients were approached with 33 patients recruited (11 Control, 10 Education, and 12 Education+Feedback). Retention at 2-week follow-up was 52% (17/33; n=6 for Control, n=3 for Education, and n=8 for Education+Feedback). No study-related adverse events occurred. Collectively, patients responded to 106 out of 325 vibration prompts from the waist-worn inclinometer (32.62%). Within 5 min of the prompt, 41% of responses occurred, with patients standing for a mean 1.4 (SD 0.8) min and walking for 0.4 (SD 0.3) min (21, SD 11, steps). Interviews indicated that being unwell and overwhelmed after an exacerbation was the main reason for not engaging with the intervention. Health care staff considered reducing sedentary behavior potentially attractive for patients but suggested starting the intervention as an inpatient.

**Conclusions:**

Although the data support that it was feasible to conduct the trial, modifications are needed to improve participant retention. The intervention was acceptable to most patients and health care professionals.

**Trial Registration:**

International Standard Randomized Controlled Trial Number (ISRCTN) 13790881; http://www.isrctn.com/ISRCTN13790881 (Archived by WebCite at http://www.webcitation.org/6xmnRGjFf)

## Introduction

Postacute exacerbation interventions for people with chronic obstructive pulmonary disease (COPD), including pulmonary rehabilitation within 4 weeks of discharge, have been found to reduce COPD-related readmissions [1]. An acute exacerbation is characterized by a “sustained worsening of the patient’s condition, from the stable state and beyond normal day-to-day variation that is acute in onset and may warrant additional treatment in a patient with underlying COPD” [2]. Despite the benefits, postdischarge pulmonary rehabilitation is sparsely taken up at the point of discharge (9.6% of all hospital discharges) [3]. One reason for this may be that increasing physical activity or exercise can be a daunting prospect for many patients and may seem counterintuitive to managing their breathlessness [4]. Therefore, an intervention aiming to reduce patients’ sedentary behavior when they return home from hospital may be more relevant than exercise for some individuals with COPD [5,6]. In turn, this may act as a stepping stone in helping patients prepare for pulmonary rehabilitation. Sedentary behavior is defined as “any waking behaviour characterized by an energy expenditure ≤1.5 metabolic equivalents, while in a sitting, reclining or lying posture” [7]. It is currently unknown whether targeting reductions in sedentary behavior at home immediately following discharge from hospital following an acute exacerbation is feasible and acceptable to individuals with COPD.

Wearable technology may help patients engage with their health [8], and although there is evidence suggesting that wearables, such as pedometers, help individuals with COPD to increase their physical activity [9,10], no studies have specifically targeted sedentary behavior. Haptic feedback provided as vibration prompts has been used successfully in a range of contexts, including sports coaching [11], gait and balance training for older adults [12], and learning new skills [13]. The use of vibration prompts in behavior change interventions is gaining momentum and has been found to be an acceptable approach to reducing sitting time in sedentary men [14]. The Chronic Obstructive Pulmonary Disease Sitting and ExacerbAtions Trial (COPD-SEAT) aimed to examine the feasibility of the trial and acceptability of the intervention to reduce sedentary behavior at home in patients with COPD following hospitalization for an acute exacerbation. Furthermore, we interviewed patients and health care professionals involved in COPD exacerbation care to understand their perspectives of reducing patients’ sedentary behavior in this context.

## Methods

### Design

The study design was a 3-armed feasibility randomized controlled trial (RCT) lasting 2 weeks following discharge from hospital, with 1:1:1 allocation. A detailed description of the study protocol has been published previously [15].The trial is reported in accordance with CONSORT-EHEALTH (Multimedia Appendix 1). The study was approved by Research Ethics Committee East Midlands Leicester Central and all participants provided written informed consent (15/EM/0433).

### Recruitment

Individuals admitted to Glenfield Hospital (Leicester, UK), between February and June 2016, were screened for eligibility by COPD Specialist Nurses. Inclusion criteria were: aged 40 to 85 years; confirmed COPD diagnosis as described in patient notes; confirmed acute exacerbation of COPD as the reason for hospitalization; fewer than 4 exacerbations requiring emergency admission to hospital in the previous year, and deemed by the COPD Specialist Nurses to be physically able to participate in light-intensity physical activity. Patients were invited to take part during their hospital stay, face-to-face by a study researcher (MO), after being seen by a COPD Specialist Nurse as part of usual care. Eligible patients were given a verbal description of the study, participant information sheet, and expression of interest form. The researcher revisited patients at the bedside at an agreed time to collect the expression of interest. For patients wishing to take part in the study, written informed consent was obtained. The timing of these procedures varied based on expected discharge. Participants were not required to have access to the Internet to take part and were not paid for taking part.

### Randomization

Block randomization was conducted using sequentially numbered sealed envelopes by an individual independent of the research team. Due to limited study team members and logistical barriers, researchers were made aware of group allocation before obtaining consent. Patients were informed of their group allocation after providing informed consent.

### Intervention and Control Groups

Patients were randomized in-hospital to one of the 3 groups: “Control,” “Education,” or “Education+Feedback.” Study interventions were delivered in-hospital (face-to-face) by a researcher (MO). The Education group received verbal and written information about reducing sedentary behavior in the form of a booklet entitled *Sit Less, Move More, Live Healthier*, adapted for COPD from *On Your Feet to Earn Your Seat* [16,17]. The researcher went through the material with each participant at the hospital bedside, discussing the importance of breaking up prolonged sitting and how this could be done at home. The booklet contained 7 main suggestions: leave the house daily; make advertisement breaks active; stand-ups (eg, when the kettle is boiling); tiptoe through the queue; increase your steps; sit to stand with no hands; and treat the seat as a treat. The Education+Feedback group received the same educational component plus real-time feedback on their step count, sitting, standing, lying down, and sit-to-stand transitions via an inclinometer linked to a smart device application provided for them. Additionally, these patients received haptic feedback (vibration prompts) from the inclinometer when they were sedentary for a prolonged period of time. This feature was modified from the original purpose of the device, which was to vibrate when the user was in poor posture. The timings of the vibration prompts (eg, after 30 min of sitting) were determined by the patient in-hospital. The setting of how long patients could be sedentary for before the prompt could not be altered after patients were discharged from hospital (eg, they could change from 30 min to 40 min). No changes to the education booklet, inclinometer, or smart device application were made during the study. Patients took part in the interventions for 2 weeks following discharge and were not prompted to engage with the intervention during that time.

All patients in the trial received the discharge bundle as part of usual care (control condition). The care bundle comprised advice about doing regular exercise (no actual supervised exercise conducted with patients), attending pulmonary rehabilitation, medication advice, inhaler training, mobility physiotherapy input, and in-hospital physical function discharge assessments [18]. Patients were provided with telephone contact details of the COPD Specialist Nurses.

### Feasibility of the Trial

#### Recruitment and Retention

Patient eligibility, uptake, and retention were recorded. Patients not wishing to take part were asked for their main reason for this. To monitor intervention safety, adverse events were recorded for each patient during their time in the study. Readmissions for an acute exacerbation were not considered adverse events as up to 43% of patients may be readmitted within 3 months in any case [19]. Descriptive characteristics were compared between those who did and did not attend the 2-week follow-up appointment.

#### Intervention Fidelity

Deliveries of the Education and Education+Feedback components of the intervention were audio recorded. Details of the intervention components (education booklet, application, and vibration prompts) have been previously reported [15]. Each component was coded separately by 2 trained, independent assessors using dichotomous scales (present or absent) for consistency and ordinal scales (poor, adequate, or excellent) for quality of delivery.

#### Dropout Interviews

Dropout telephone interviews were conducted to explore reasons why patients did not attend the follow-up appointment. The questions asked included what they thought of the study, how they were feeling and what had been going on during the study, and their reasons for withdrawal.

#### Health Care Professional Interviews

Semistructured interviews with doctors, COPD Specialist Nurses, ward nurses, and physiotherapists were conducted to examine the perspectives of staff involved in the COPD care pathway and provided insights on suggestions for and the potential barriers of conducting a full-scale RCT. They were asked about their thoughts on the intervention itself, reducing sedentary behavior for this patient population, and the education material and technology.

### Acceptability of the Intervention

#### Wear Adherence, Charging Compliance, and Missing Data

All participants were asked to wear the inclinometer for 14 consecutive days, with discharge date considered as day 0. The number of days the inclinometer was worn by participants was examined. One overnight charging occurrence per 24 hours (13 in total) was considered 100% charging compliance. Charging was automatically detected by the inclinometer. Patients were asked to charge the inclinometer every day to remove the need for patients to check the battery status manually. The battery life of the inclinometer typically lasts for 2 to 3 days before charging is required. Missing data were examined to determine whether this was caused by a depleted battery or from being manually switched off.

#### Engagement With Smart Application

Patient engagement with the LUMOback app was quantified using Flurry Analytics [20], which registered, offline, each swipe and tap performed by patients for the sitting time, stand-ups, and step count panels.

#### Responses to Vibration Prompts

**Figure 1:**
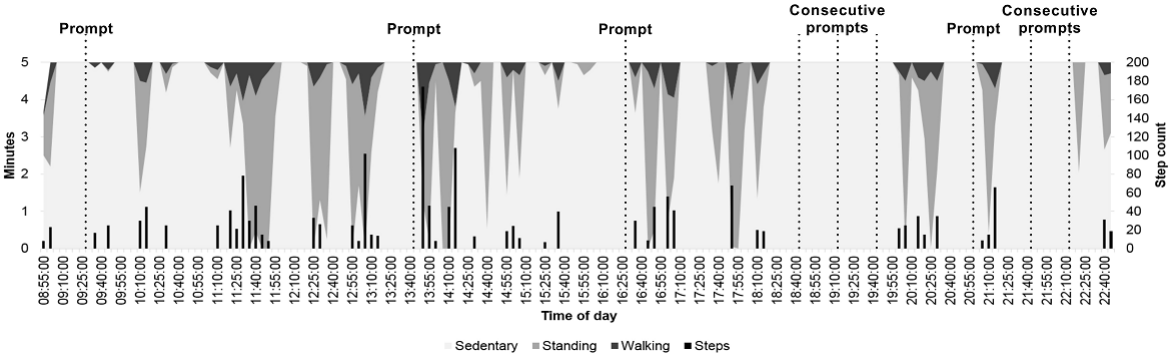
A concept diagram demonstrating where vibration prompts (set for 30 consecutive minutes sedentary) would occur during the course of a day. The dashed lines indicate where a vibration prompt would have occurred. The darker shaded areas depict where a patient has interrupted their sedentary time with standing and/or walking, and the black bars represent step count. Prompts at 9:30, 14:45, 16:30, 21:00 and 22:15 are followed by physical activity within 5 min of the prompt taking place. Prompts at 18:45, 19:15, 19:45 (3 consecutive prompts) and 21:45 are not followed by physical activity.

Vibration prompt identification was based on periods of consecutive time spent sedentary as per patients’ choice of vibration setting. After time-stamped vibration prompts were identified, the subsequent 15 min were analyzed to examine whether patients responded to the vibration prompt and, if they did, how long it took and what they decided to do. A concept diagram using real data for when vibration prompts occurred is provided in Figure 1. From the figure, it can be seen that prompts occurring at 9:30, 14:45, 16:30, 21:00, and 22:15 were followed by physical activity within 5 min of the prompt taking place, whereas 3 consecutive prompts at 18:45, 19:15, and 19:45 and a prompt at 21:45 were not followed by physical activity.

#### End-of-Study Interviews

Semistructured interviews with patients in the intervention groups were conducted during the follow-up appointment at Glenfield Hospital, United Kingdom. Interviews explored patients’ experiences of the self-monitoring technology, vibration prompt, application, education booklet, and the study overall.

### Attendance at Pulmonary Rehabilitation

Past and present pulmonary rehabilitation referral and attendance information was obtained through the Hospital Information Support System on-site at Glenfield Hospital, United Kingdom. The proportion of patients attending the pulmonary rehabilitation clinic assessment as part of usual postexacerbation care, the proportion of patients who agree to take part, and the proportion of patients to go on to attend are reported.

### Sample Descriptive Measures

Measures were obtained during the hospital stay to describe the study sample. Symptom burden was self-reported using the COPD Assessment Test [21] and usual modified Medical Research Council dyspnea grade [22]. Fatigue was self-reported using Functional Assessment of Chronic Illness Therapy-Fatigue with a score <30 indicating severe fatigue [23]. Anxiety and depression were examined using the Hospital Anxiety and Depression Scale [24] with a normal score considered as 0 to 7, borderline abnormal level considered 8 to 10, and abnormally high anxiety/depression considered 11 to 21 [25]. Fear of falling was self-reported by Falls Efficacy Scale-International (0-64, with higher score denoting more fear of falling) [26]. Self-reported usual time spent sitting was obtained from the Marshall Sitting Time questionnaire [27]. Information on the ownership and usage of computers and smartphones was self-reported. Index of Multiple Deprivation, which ranks the relative deprivation of postcodes in England was used. At the follow-up appointment, height, weight, and waist circumference were measured. Body mass index (BMI) was calculated, and patients were categorized as either underweight (<18.5 kg/m^2^), normal weight (≥18.5 and <25.0 kg/m^2^), overweight (≥25.0 and ≤30.0 kg/m^2^), or obese (≥30.0 kg/m^2^) [28]. Patients completed the short physical performance battery (SPPB) with a score <10 points considered the threshold for mobility limitation [29].

### Assessment of Physical Activity and Stationary Time

Participants were asked to wear an ActiGraph wGT3X-BT accelerometer (ActiGraph, Pensacola, FL) on the right anterior hip during waking hours for 14 days following discharge (full methodology in Multimedia Appendix 2). Steps and intensity of physical activity undertaken during the study period were reported with stationary time classified as <100 counts per min [30]. A valid day was considered ≥8 hours of valid waking wear time, with patients providing ≥4 valid days out of 7 for both weeks included in the analyses [31].

### Quantitative Analyses

Comparisons between groups and between patients who did or did not complete the study were performed using independent *t* tests and analysis of variance. Categorical data were analyzed using chi-square (n≥5) or Fisher exact test (n<5). Analyses were conducted using SPSS version 22.0 for Windows (SPSS Inc., Chicago, IL), with alpha set to .05. The datasets used and/or analyzed during this study are available from the corresponding author on reasonable request.

### Qualitative Analyses

Interviews were conducted by a trained social scientist (AW), audio-recorded, and transcribed. Transcripts were analyzed initially by AW, for themes pertinent to the feasibility of the study and acceptability of the intervention [32], using constant comparison [33] and facilitated by NVivo 10 qualitative software (QSR International, Cambridge, MA). Themes were discussed against the material with PS and JD.

## Results

### Feasibility of the Trial

#### Eligibility, Uptake, and Retention

**Figure 2:**
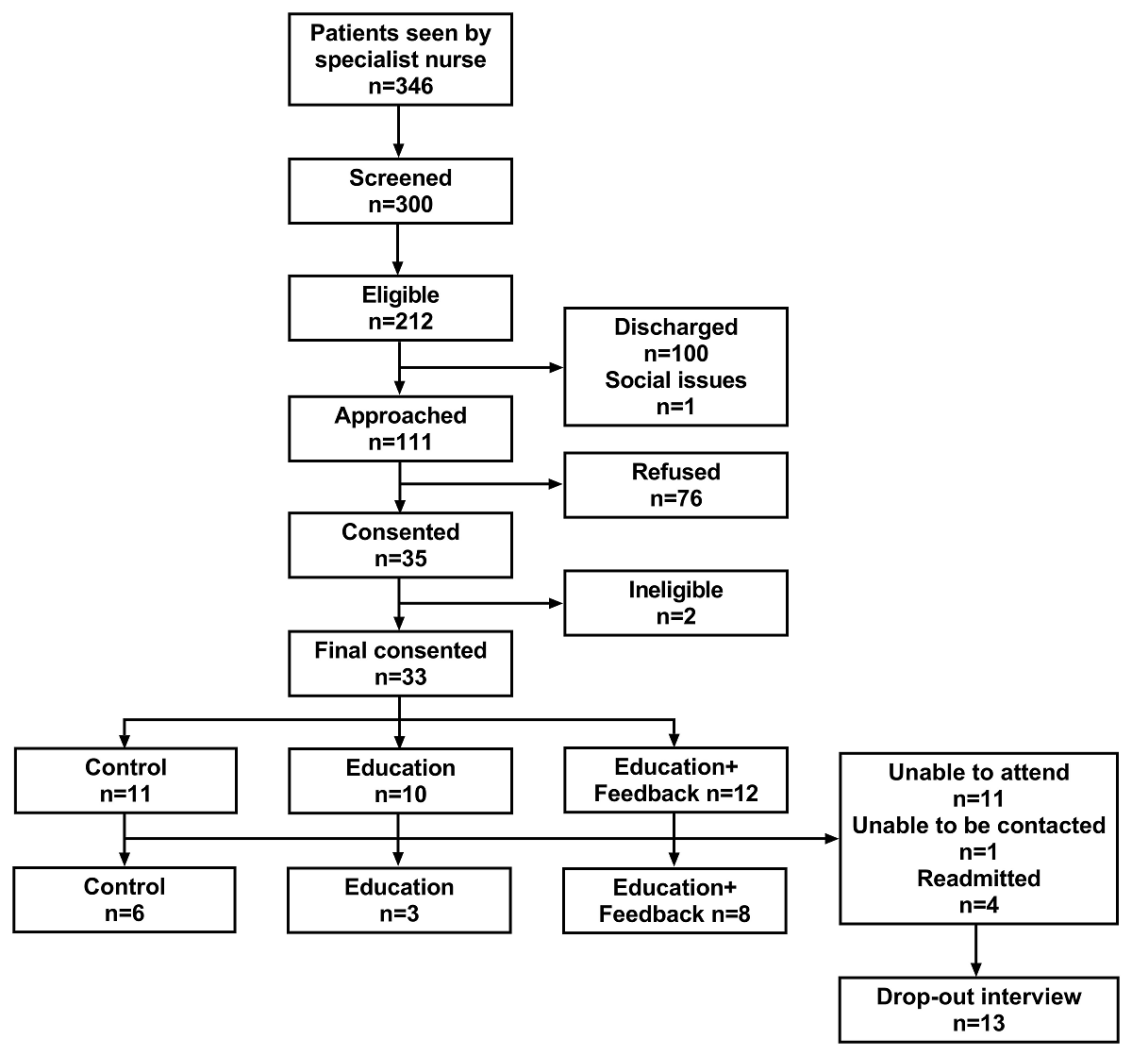
Consolidated Standards of Reporting Trials (CONSORT) diagram.

Participant flow through the trial is presented in Figure 2. Of the 300 patients screened, 212 (70.7%) were eligible to take part in the study. Of these, 100 (47.2%) were discharged before the researcher could approach them, and the study team were advised not to approach one patient because of a complicated social situation. Finally, 111 patients (52.4% of eligible patients) were approached to take part in the study, with 35 (31.5%) consenting to participate. However, 2 patients were identified as having early-stage dementia, leaving 33 patients (11 Control, 10 Education, and 12 Education+Feedback). Of these, 17 (51.5%; 6 Control, 3 Education, and 8 Education+Feedback) attended the follow-up appointment. Rate of recruitment averaged 2.2 patients per week.

#### Patient Characteristics

The sample comprised mostly female (23/33, 70%), retired (25/33, 76%), former smokers (21/33, 66%) who self-reported sitting for an average of 9.2 (SD 4.2) hours/day (Table 1). The majority (25/33, 76%) of participants did not own a smartphone but did own a computer (20/33, 61%). Of the total sample, 30% of patients (10/33) were classified as having abnormally high depression scores, 39% (13/33) were classified as having abnormally higher anxiety scores, and 81.8% (27/33) of patients were classified as having severe fatigue. All patients who completed the study completed all questionnaires. On the basis of follow-up data of patients who completed the study, 65% of patients (11/17) were overweight or obese, 6% (1/17) were underweight, and 96% (16/17) had mobility limitation (SPPB <10 points). Control completers had significantly greater BMI and waist circumference than Education and Education+Feedback completers (eg, 21.3, SD 3.2, vs 37.3, SD 9.7, vs 28.8, SD 3.8 kg/m^2^, respectively). Noncompleters had higher levels of postcode deprivation and had more readmissions during the study period than those who completed the study.

**Table 1:** Patient characteristics for the whole sample and stratified by attendance at the 2-week follow-up appointment. COPD: chronic obstructive pulmonary disease.

Descriptor		Whole sample (N=33)	Completed (N=17)	Not completed (N=16)
Age in years, mean (SD)		71.0 (20.0)	66.6 (9.6)	69.5 (11.2)
Female sex, n (%)		23 (70)	12 (71)	11 (69)
Index of Multiple Deprivation decile, mean (SD)		4.5 (3.2)	5.8 (3.1)	3.0 (2.6); *P*=.008
**Employment status, n (%)**				
	Retired	25 (76)	12 (71)	13 (81)
	Unemployed	5 (15)	4 (24)	1 (6)
	Employed	3 (9)	1 (6)	2 (13)
**Smoking status^a^, n (%)**				
	Current	11 (34)	4 (24)	7 (44)
	Former	21 (66)	12 (71)	9 (56)
Pack years, mean (SD)		46.7 (25.6)	44.3 (26.5)	49.5 (25.1)
Usual modified Medical Research Council grade, mean (SD)		2.6 (1.2)	2.7 (1.1)	2.6 (1.3)
Home oxygen, n (%)		3 (9)	2 (12)	1 (6)
Number of comorbidities, mean (SD)		3 (3)	2 (3)	4 (3)
Acute exacerbation of COPD readmissions, n (%)		4 (12)	0 (0)	4 (25); *P*=.043
COPD assessment test score, mean (SD)		24.9 (7.5)	24.5 (7.4)	25.4 (7.8)
Fatigue score, mean (SD)		19.8 (11.8)	21.0 (12.2)	18.1 (11.7)
Hospital Anxiety and Depression Scale depression score, mean (SD)		8.2 (4.7)	7.4 (5.2)	9.3 (3.9)
Hospital Anxiety and Depression Scale anxiety score, mean (SD)		9.2 (5.8)	8.9 (5.5)	9.6 (6.2)
Falls Efficacy Scale-International score, mean (SD)		33.4 (13.7)	31.5 (13.5)	35.4 (14.2)
Self-reported daily sitting time in min/day, mean (SD)		553.0 (253.6)	603.1 (257.4)	487.9 (245.9)

^a^Missing n=1.

#### Reasons for Ineligibility and Nonparticipation

The most common reasons for ineligibility were as follows: too severe comorbidities (36.4%), more than 4 exacerbations in the previous year (20.5%), and taking part in other research (14.8%). The most common reasons for not taking part in the study were as follows: feeling too unwell or having too many health-related issues/commitments (40.0%) and considering themselves sufficiently active (12.9%). Two patients (2.4%) were put off by the activity monitors.

#### Readmissions and Adverse Events

Two patients (6%; Education) were readmitted to hospital for at least one overnight stay for respiratory (n=1) and nonrespiratory (n=1) issues. Although not considered an adverse event in this study, 4 patients (12%; 1 Control, 3 Education+Feedback; withdrawn from the study) were readmitted for an acute exacerbation of COPD during the 2-week follow-up. No hospital admissions were study-related, and no participants died during the trial.

#### Intervention Fidelity

We delivered 21 (95%) interventions as planned. However, one intervention (Education) was not delivered verbally because of the patient being discharged. A full breakdown of intervention fidelity is provided in Multimedia Appendix 3. Overall consistency of the intervention delivery was 77.3%, with 0.2%, 9.4%, and 90.4% for “poor,” “good,” and “excellent” quality ratings, respectively.

#### Reasons for Patients Dropping out of the Study

When 13 participants (81% of those who dropped out) were asked about their reasons for dropping out of the study, patients stated they were too unwell and overwhelmed after experiencing an exacerbation, sometimes dealing with comorbidities (eg, urinary tract infection and heart failure), medications, hospital appointments, readmission for another exacerbation, and lack of support from friends and family (Textbox 1). When asked what would help them to sit less, patients responded with answers such as “getting better.” Patients did not suggest any specific changes to the study and were often disappointed or apologetic with not being able to give it a go, indicating they would have liked to try the intervention at a later date when symptoms are more stable.

#### Views of Health Care Professionals

Health care staff reported in interviews that decreasing sedentary behavior would be a good way to increase patients’ activity levels and prevent them from being readmitted (Textbox 2). Although a few thought the timing of the study was good because it encouraged movement at home, many suggested starting the intervention during the patients’ hospital stay because it would help motivate them to move in hospital, where they often became bedbound. Staff also noted that they could help patients get used to the device during hospital stay but also remarked that this would increase workload. Nearly all the health care staff felt that the technology was a good idea, as it would give patients something to focus on and encourage them to sit less. However, staff noted that the patients, the majority of whom were older adults, might have limited technological ability and that the more severely ill patients might find the concept overwhelming.

### Acceptability of the Intervention

#### Wear Adherence, Charging Compliance, and Missing Data

Patients wore the inclinometer for 11.8 (SD 2.3) days over the 14-day period. Of those who participated, 67% of the patients charged the device on ≥7 days and 47% charged the device on ≥10 days; 20% of the participants experienced a device malfunction. Two types of malfunction were reported: the smart device would not turn on, which meant patients were unable to use the mobile app (the vibration prompt remained functional), and a delay in communication between the inclinometer and the app. Missing data occurred for 3 participants: 1 turned the inclinometer off for 1 day, 1 had 8 missing days (battery died for 5 days and turned off for 3 days), and 1 had 12 missing days (turned off).

#### Engagement With Smart Application

Three patients (25%) actively engaged with the LUMOback app during the 2-week follow-up. One male patient used the app on day 1 to look at the sitting time summary (131 seconds), the stand-ups summary (3 seconds), and step count summary (6 seconds) and detailed step count information (8 seconds). This patient experienced a device malfunction on day 2. Despite a replacement sensor being provided, it was not possible to obtain further app interaction data. The other 2 patients did not engage with the app beyond the use of the summary tile automatically on show when unlocking the smart device (unknown durations as no swipes or taps on the screen occurred).

#### Responses to Vibration Prompts

**Figure 3:**
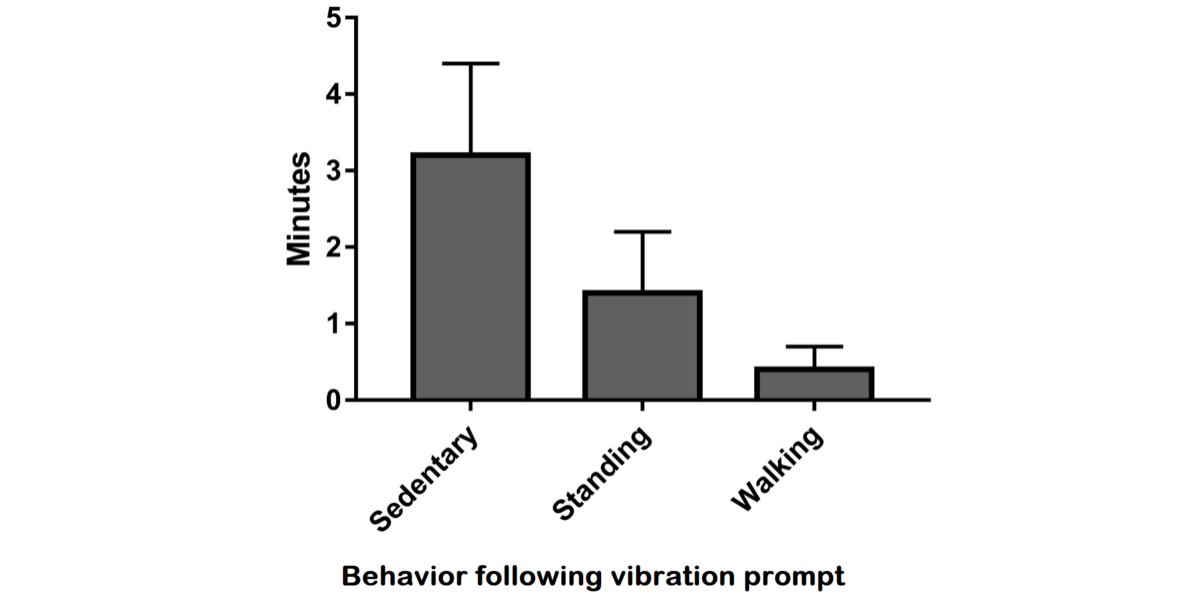
Behavioral responses to the vibration prompt for the 5 min following the prompt occurring.

Of the 12 patients randomized to the Education+Feedback group, 6 chose for the vibration to occur after 30 min (4 completers), 1 after 45 min (1 completer), and 5 after 60 min of consecutive sitting (3 completers). Collectively for the 8 patients who completed the study, 325 vibration prompts occurred. Patients did not respond to 67% of the prompts. When patients did respond to the prompts, 40.6% responses occurred within 5 min of the prompt, with patients spending 1.4 (SD 0.8) min standing and 0.4 (SD 0.3) min walking, taking 21.2 (SD 11.0) steps (Figure 3).

**Patient panel: illustrative quotes from patient dropout interviews.**

“I am really disappointed, but it’s just the way it is, I have so much on my plate right now, and the heart failure thing really knocked me.” [Female, Education 009]

“I had an awful lot of hospital appointments and doctors’ appointments, and it was like. Do you know what, I’m getting fed up with this. It was too much for me, I just wanted to relax and get better.” [Female, Education 021]

“I’ll try again, I want to do it, but maybe when I feel a bit better.” [Female, Education 027]

**Health care panel: illustrative quotes from health care qualitative interviews about the feasibility of the trial.**

Views on study design

- “I think that’s a really good idea. It will give them something to focus on. It may give them a bit of drive, a bit of focus, and it encourages people to become active, cuz obviously a lot of these patients who get very breathless and they can’t walk too far, so that will prompt them just to do small bits of exercise.” [HC020]
- “They are looking forward to getting home and getting back to some kind of normality, but hopefully they are thinking to themselves, I don’t wanna do that again anytime soon, so what’s gonna help me not do that again, and this study could help with that.” [HC011]

Perspectives on timing

- “There’s nothing wrong with the timing it’s just the amount of information, I think, because when you’re not well yourself and to be bombarded with a lot of information to take on board you’re sitting there listening but how much do you take in.” [HC006]
- “Round the ward would be good to encourage them to move, but if it was in the ward, the limitation would be those who can’t move without our help, and we don’t have enough nurses to go to bed 1 or 2, but it would help them regain confidence before going home.” [HC021]

Views on technology

- “I think it would be a good prompt to remind them to move and exercise, and it might help them focus, I think it’s a good idea.” [HC026]
- “Some patients, maybe the older ones possibly who are just not very technologically savvy, they may struggle with something like this, and maybe the ones who are more end stage COPD, they might not see the point or be too overwhelmed possibly.” [HC022]

The proportion of days prompted were similar between patients who chose a 30-min prompt setting and patients who chose a 60-min prompt setting (76% and 77% of days, respectively). Patients who chose the 30-min setting were prompted more frequently than those who chose the 60-min prompt setting (5.5 and 1.6 prompts per day, respectively), but the proportion of nonresponses and responses within 5 min of the prompt occurring were similar (53% vs 59% nonresponses and 19% vs 23% responses within 5 min, respectively).

#### Views of Patients Who Completed the Study

Three themes pertinent to the acceptability of the intervention were identified from the interviews: (1) being too unwell and overwhelmed, (2) engaging with the intervention when it fitted with routines, and (3) perspectives on leaflet and wearable technology. Supporting quotes for the themes identified are provided in Textbox 3.

##### Being Too Unwell and Overwhelmed

Many patients still felt unwell after their exacerbation and talked about being too overwhelmed with hospital appointments and medications to be able to fully engage with the intervention. They discussed about struggling to do normal daily activities and not engaging with the study intervention but instead preferred sitting and relaxing to make themselves feel better. A few patients were readmitted for their COPD within a week of their discharge, and a few also experienced newly diagnosed or ongoing comorbidities. Patients talked about the study not being their priority while illnesses and treatments were on their agenda.

##### Engaging With the Intervention When It Fitted With Routines

Patients who engaged with the intervention described that they typically reduced their sitting time when it fitted with their routines. For example, they responded to the vibration prompt by getting up to make tea or walk the dog. When the prompts interrupted an activity deemed important/enjoyable to the patient, such as watching an interesting program on TV, the prompt was experienced as disruptive and annoying.

A couple of patients reported adopting new routines, such as walking to the bottom of the garden in response to the vibration. Several participants mentioned that they made more of an effort to reduce their sedentary time in the second week rather than the first, when they felt better.

##### Leaflet and Technology Use

Most participants found the technology fairly easy to use and wore and interacted with it. With regard to the educational component, many did not recall the leaflet, some mentioned they had read but forgotten it, or felt that it was not relevant or useful to them, and many said they did not really sit anyway and they found the tips were not applicable for them. However, some patients mentioned that they had read it and it made them think about sitting, and they thought the overall concept was a good idea. Many said the waistband was uncomfortable and suggested that a wristband would have been easier to use. The study in general appealed to most participants, with many saying they had good intentions and considered that they might gain some knowledge from it for themselves and others.

#### Pulmonary Rehabilitation

Of the 33 patients who took part in the trial, 14 (42%) attended the clinic assessment, 7 (21%) agreed to attend, and 4 (12%) went on to attend pulmonary rehabilitation (Table 2); 9% of patients in the Control group, 10% of patients in the Education group, and 17% of patients in the Education+Feedback group went on to attend pulmonary rehabilitation.

**Patient panel: illustrative quotes from patient qualitative interviews.**

Too unwell and overwhelmed

- “I’ve had to sit a lot. There’s a lot going on with my health, and I just can’t cope sometimes. I’m struggling with even my normal stuff.” [Female, Feedback 017]
- “You need so much energy to get through the day, it’s difficult when you get home, and you’re trying to recover and getting up is sort of difficult then, you just want to sit and relax and get better.” [Female, Feedback 026]

Fitting with routine

- “You feel it buzz on your back, so I just get up and walk in the kitchen or go and put the kettle on.” [Male, Feedback 010]
- “It does give you a sense of purpose, you know, it goes off and you walk the dogs or go round to the neighbours or something like that. It clocks it up.” [Male, Feedback 010]
- “I was annoyed that this thing was poking me in the back every half an hour, cause I didn’t want to move, I was watching something.” [Female, Feedback 020]
- “The first week was dreadful, I just wasn’t feeling myself. Anyway the next week I started feeling a lot better, started doing my normal things again.” [Female, Feedback 029]

Leaflet and technology use

- “It [the device] was fine. No problem, fairly easy to use really. I put it on in the morning and left it on all day until I went to bed at night.” [Female, Feedback 020]
- “I read it [the leaflet]. Forgot it. It didn’t really have anything in it that interested me, I don’t watch much TV or anything.” [Female, Education 012]
- “It’s been a bit uncomfortable, because it’s been hot, you know, and I couldn’t put any thin trousers on because I’m wearing it, but it’s been alright, yeah. Maybe a wrist thing would have been better for me.” [Female, Feedback 029]
- “I think it’s a good study, it gives insight, doesn’t it, to other people and for me too really.” [Female, Education 008]

**Table 2:** Uptake to pulmonary rehabilitation (PR) stratified by study group.

Stage of pulmonary rehabilitation	Whole sample (N=33)	Control (N=11)	Education (N=10)	Education+Feedback (N=12)
Attended clinic assessment, n (%)	14 (42	8 (73)	3 (30)	3 (25)
Agreed to take part in PR, n (%)	7 (21)	3 (27)	2 (20)	2 (17)
Attended PR, n (%)	4 (12)	1 (9)	1 (10)	2 (17)

#### Changes in Physical Activity and Stationary Time

**Figure 4:**
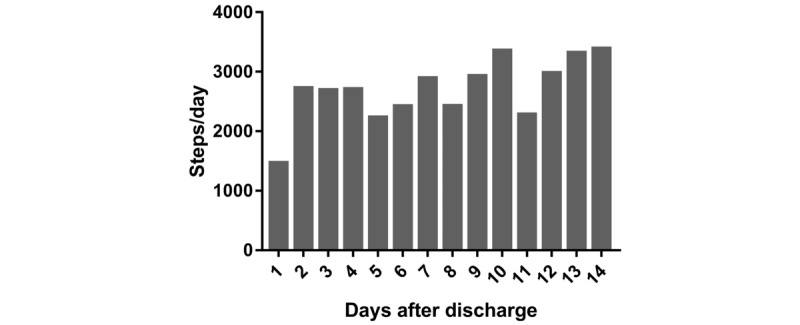
Daily step count during the 14-day study period for patients with valid data (≥4 day out of a possible 7 for each of the 2 weeks). N=8 for day 7 and day 8; N=7 for day 2, day 3, day 4, day 9, day 12, and day 13; N=6 for day 1, day 5, day 6, and day 11; and N=5 for day 10.

**Figure 5:**
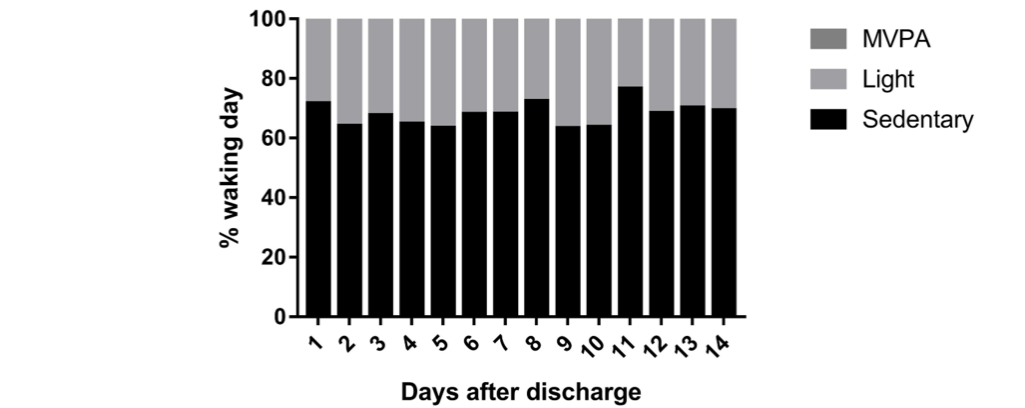
Proportion of each waking day spent being stationary, in light activity and in moderate to vigorous physical activity (MVPA) during the 14-day study period for patients with ≥4 valid days for each of the 2 weeks. N=8 for day 7 and day 8; N=7 for day 2, day 3, day 4, day 9, day 12, and day 13; N=6 for day 1, day 5, day 6 and day 11; and N=5 for day 10.

A total of 14 patients (42%) provided at least 1 day of valid accelerometer data for both the first and second week post discharge reducing to 5 patients (15%) providing 6 valid days for both the first and second week post discharge (total 12 days). On the basis of 8 patients (24%) who provided at least 4 days of valid accelerometer data for both the first and second week post discharge (total ≥8 days; 3 Control, 1 Education, 4 Education+Feedback), step count increased on average by 1920 steps per day (+43%) from the first day after discharge to day 14 (Figure 4). The proportion of stationary time, light activity, and moderate to vigorous physical activity did not change over the 2-week period, with stationary time ranging from 63% to 77% and MVPA ranging from 0.3 to 0.9% of patients’ waking day (Figure 5).

#### Changes in Self-Reported Health Questionnaires

A comparison of baseline versus 2-week follow-up responses with health questionnaires for patients with complete data (6 Controls, 3 Education, and 8 Education+Feedback) is provided in Multimedia Appendix 4. No statistically significant changes over time were observed for all groups for COPD symptoms (COPD Assessment Test), fatigue, anxiety, depression, or fear of falling (Falls Efficacy Scale-International score). The proportion of patients reporting severe fatigue reduced from 71% to 41% for the whole sample over the 2 weeks, which can be attributed to the natural postexacerbation recovery of patients.

## Discussion

### Principal Findings

This study examined trial feasibility and the acceptability of an education and self-monitoring intervention using wearable technology to reduce sedentary behavior for individuals with COPD hospitalized for an acute exacerbation. Approximately one-third of patients who were offered the study took part, and of these, around half attended their follow-up appointment at 2 weeks. Reasons for deciding not to take part were predominantly being too unwell or being readmitted. Patients responded to approximately one-third of the vibration prompts provided by the wearable technology, of which approximately 40% occurred within 5 min, resulting in approximately 1.5 additional min standing, approximately 0.5 min of walking, and approximately 21 extra steps per response.

### Feasibility of the Trial

The trial seems feasible with the proportion of eligible patients (70.7%) comparable with early pulmonary rehabilitation (63.8% eligible) [3]. This study’s uptake (31.5%) was also similar to that of a periexacerbation pulmonary rehabilitation study (32.3%) conducted in the same hospital [34], and the rate of recruitment was faster than a physical activity intervention using wearable technology (Fitbit Ultra) at a similar time point (average of 2.2 vs 0.6 patients per week) [35]. Retention of participants to the 2-week follow-up appointment (51.2%) was lower than the proportion of patients completing early pulmonary rehabilitation (71.7% of patients attending rehabilitation) [3]. However, for patients in the Education+Feedback group, retention (75%) was comparable to a previous behavioral telehealth intervention in this population [36], suggesting that receiving haptic feedback did not contribute significantly to the observed attrition. The relatively poor retention rate overall may be related to the length of the follow-up. When patients are discharged from hospital, symptoms remain elevated and it takes time for patients to return (or get close) to their normal activities and symptom severity [37,38]. The qualitative dropout and end-of-study interviews illustrated that many participants struggled to engage with the intervention immediately after being discharged because of multiple issues related to coping with health, which was also intimated by some of the health care staff, who suggested offering patients more support with the intervention during the hospital stay.

The recruitment method was only able to invite a proportion (47%) of admitted patients to take part in the study. This was because patients were discharged between screening and approach. Usual care, required to determine eligibility and conducted by COPD Specialist Nurses, was provided to all patients. With pressure for wards to discharge patients and variable patient recovery rates, this usual care cannot always be conducted in the early days of admission. In addition, 45% of patients are admitted and discharged within 3 days [39]. Therefore, with 1 person recruiting for this study (after usual care was completed), it was not always possible to approach eligible patients before they were discharged. Additional opportunities for patients to engage with lifestyle program may be warranted. For example, the future iteration of this trial could offer multiple opportunities for patients to take part when they feel well enough, including during their hospital stay, once they have returned home, and before attending pulmonary rehabilitation. If in the next trial it is not possible to approach patients during their hospital stay, patients could be sent study information by post and contacted via telephone. The challenge for future work will be to maintain contact with the patient and intervene regardless of whether or not the patient is still in hospital. This is because the discharge date is often influenced by other factors such as the need for family or social support.

### Acceptability of the Intervention

Overall, participants adhered to wearing and charging the waist-worn inclinometer. However, with an increasing recognition of the importance of capturing the 24-hour day for understanding behavior and health outcomes [40], there is a need to shift the traditional locations for activity monitoring (eg, the waist) toward locations facilitating better compliance (eg, the wrist). Interviews highlighted that most patients found the inclinometer easy to use and were more engaged with it than the educational booklet and app; however, several noted that they found the waist location uncomfortable. Therefore, future work should consider moving activity monitoring to a more desirable location on the body.

Participants rarely talked about the usefulness or relevance of the specific components of the education booklet, although some mentioned they did not find some of the tips relevant as they did not relate to their habitual activities, for example, they did not watch television. Matei and colleagues [17] observed good adherence to most suggestions (eg, 61% for “making ad breaks active” and 55% adhering to “leave the house daily”) in older adults recruited from sheltered housing sites. Other tips were adhered to less, with 15% adhering to “tiptoe through the queue” [17]. This highlights the need to individually tailor the education component to the specific sedentary behaviors of patients and to capture adherence rates to the education components in future work.

In addition, the lack of engagement with the app may reflect the additional effort required to use unfamiliar technology (only 24% owned a smartphone) and, as described by patients during the interviews, to comprehend and act on information at a time when they were still unwell and struggling to cope with their COPD and comorbid conditions. This study did not offer training on using the smart device, which may have contributed to the poor engagement with the app. The next iteration of the trial should account for the variability between patients in their confidence and ability to use technology. With time, a greater proportion of patients are likely to own smart devices and be more comfortable using such technology.

The proportion of positive behavioral responses to the vibration prompts and the resulting additional physical activity were promising (eg, 21, SD 11, extra steps per positive response). It is perhaps unrealistic to expect patients to respond to all vibration prompts as some of the unheeded nudges may have been the result of poor timing as the technology was not “context aware.” For example, prompts could have gone off while a patient was in their car. The interviews illustrated that patients often responded to the prompts when this naturally fitted with their routines; furthermore, some chose to ignore the vibration when they interrupted enjoyable activities. In this respect, our participants did not easily introduce new behaviors, as in the study by Matei and colleagues [17] focusing on healthy older adults and offering tips to reduce sedentary time. This may have been because patients in this study had a chronic condition, were acutely unwell, and had returned home following hospitalization, but may have also been because they were asked to engage with a wearable monitor that interrupted everyday routines. Patients chose how long they could be sedentary before being prompted, with half choosing to be prompted after 30 min of sitting. The range in patient preferences of vibration prompt occurrence supports future work offering this choice to patients. However, the unstandardized prompt frequencies between patients must be accounted for in the future efficacy trial. Due to usage restrictions placed on the smart device, the choice of vibration setting could not be changed during the 14 days. Additional flexibility permitting patients to alter the frequency of the feedback may facilitate greater engagement with the intervention in the context of their recovery, as highlighted in the qualitative interviews.

### Pulmonary Rehabilitation Participation

Although the small sample size must be considered, the proportion of participants going on to attend pulmonary rehabilitation was similar to what would be expected (approximately 14%) [3], but the proportion of patients attending from the Education+Feedback group (17%) was higher than attendance rates from the Education and Control groups (10% and 9%, respectively). Therefore, findings support the idea of providing a behavioral intervention post discharge as a stepping-stone approach to encouraging pulmonary rehabilitation participation.

### Strengths and Limitations

Strengths of the study include the use of qualitative and quantitative methods to examine trial feasibility and intervention acceptability, the use of novel wearable technology to provide behavior-specific nudges, asking for the perspectives of hospital staff involved in COPD care, and delivering the intervention at the bedside in accordance with the discharge care bundle. Limitations beyond those pertinent to the feasibility nature of this study included the relatively small sample size and short follow-up period, limiting the conclusions that could be drawn from statistical analyses. For example, COPD symptoms, fatigue, anxiety, depression, and fear of falling did not appear to improve over the 2-week period (however, the study was underpowered). Although we present the first intervention specifically targeting reductions in sedentary behavior for individuals with COPD, testing such an intervention in the stable state will permit an easier assessment of efficacy compared with the acute state where patients will naturally recover. No further reinforcement to attend pulmonary rehabilitation was provided during patients’ involvement in the trial and no extra support or training on the technology was provided to patients once discharged. However, these limitations are because of the “light-touch” design of the study to better reflect what future implementation may look like. Assessor and patient blinding to group allocations was not possible. The use of the ActiGraph accelerometer limited our assessment of sedentary behavior to “stationary time” rather than specific postures such as lying down, sitting, and standing. The self-monitoring technology used in this study is no longer being manufactured, an important notion to consider when examining commercially available devices and the fast-moving wearable technology industry.

### Conclusions

The data show that it is feasible to conduct a trial targeting reduction in sedentary time for individuals with COPD hospitalized for an acute exacerbation. Important areas for future work have been highlighted as follows: (1) taking a pragmatic approach of offering behavioral interventions at multiple time points; (2) improving patient stratification to identify who may need more support during behavior change interventions; (3) exploring alternative locations for objective physical activity and sedentary time intervention tools; and (4) haptic feedback using wearable technology in clinical populations. Modifications specifically required for this study include the following: (1) improved recruitment resources and methodology to approach a higher proportion of eligible patients and (2) increased flexibility of patients’ ability to engage with the intervention tool (eg, changing the vibration setting). The use of wearable technology was overall acceptable to patients and health care professionals. Responding positively to the vibration prompts resulted in meaningful increases in physical activity.

## Appendix

Multimedia Appendix 1 CONSORT‐EHEALTH checklist (V 1.6.1).

Multimedia Appendix 2 Accelerometry data collection and analytical procedures.

Multimedia Appendix 3 Intervention fidelity for education and feedback components.

Multimedia Appendix 4 Comparison of baseline versus follow-up responses to health questionnaires, stratified by study group, reported as mean (SD) unless otherwise stated.

## Abbreviations

BMI: body mass index
BRC: Biomedical Research Centre
CERS: Centre for Exercise and Rehabilitation Science
COPD: Chronic Obstructive Pulmonary Disease
COPD-SEAT: Chronic Obstructive Pulmonary Disease Sitting and ExacerbAtions Trial
NIHR: National Institute for Health Research
PR: pulmonary rehabilitation
RCT: randomized controlled trial
SPPB: short physical performance battery
