# Supplementary material for: Findings of the Chronic Obstructive Pulmonary Disease-Sitting and Exacerbations Trial (COPD-SEAT) in Reducing Sedentary Time Using Wearable and Mobile Technologies With Educational Support: Randomized Controlled Feasibility Trial
Source: JMIR Mhealth Uhealth. 2018 Apr 11;6(4):e84. doi: 10.2196/mhealth.9398 (PMC5917078; doi:10.2196/mhealth.9398)
Supplement: Multimedia Appendix 2 [file mhealth_v6i4e84_app2.pdf]

Supplementary File 1 Accelerometry data collection and analytical procedures

**Information**	**Details**
Accelerometer Model	ActiGraph wGT3X-BT (version 6.13.2; firmware 1.6.1)
Serial number range	Sixteen unique devices were used ranging from MOS2A02140493 to MOS2A02140631; averaging two deployments per device
Piezosensor orientation	Triaxial
Mode setup	Mode 29 (x, y, z, steps, lux)
Original sample rate	100 Hz (.gt3x file format)
Deployment method	Fitted by patient (on day 1) and after demonstration and practice (on day 0)
Location worn	Anterior hip adjacent to the mid-line of the thigh
Requested days of wear	14 d (20160 epochs) not including day 0
Initialization	Deployed in delay mode on day 0 (during admission) and commenced logging on day 1(first full day after discharge) at 00:00 with no stop time (estimated date of discharge not reliable)
Wear instructions	Wear continuously except for sleep and water based activities
Non-wear appropriation	≥60 min of consecutive 0s with allowance for 2 minutes of interruptions were deemed biologically implausible and coded as non-wear
Valid day criteria	≥8 hours of valid waking wear time
Valid file	≥4 valid days out of 7 days for each of the two weeks
Missing data	Data modelling or imputation was not performed
Epoch length	60 seconds
Intensity classification	Uniaxial (x-axis) cut-points as follows: Sedentary time <100 cpm; Light 100-2019 cpm; MVPA ≥2020 cpm
