# Supplementary material for: Findings of the Chronic Obstructive Pulmonary Disease-Sitting and Exacerbations Trial (COPD-SEAT) in Reducing Sedentary Time Using Wearable and Mobile Technologies With Educational Support: Randomized Controlled Feasibility Trial
Source: JMIR Mhealth Uhealth. 2018 Apr 11;6(4):e84. doi: 10.2196/mhealth.9398 (PMC5917078; doi:10.2196/mhealth.9398)
Supplement: Multimedia Appendix 3 [file mhealth_v6i4e84_app3.pdf]

Supplementary File 2 Intervention fidelity for education and feedback components

	Consistency (%)		Quality (%)		
	**Present**	**Absent**	**Poor**	**Adequate**	**Excellent**
**Education Booklet (n=21)**					
The booklet is designed to help you to sit less and move more	21 (100)	0 (0)	0 (0)	0 (0)	21 (100)
Sitting for too long can be harmful to your health	21 (100)	0 (0)	0 (0)	1 (5)	20 (95)
Use it or lose it - Keep muscles active to help prevent deconditioning	19 (90)	2 (10)	1 (5)	0 (0)	18 (95)
Other examples of benefits to be had	20 (95)	1 (5)	0 (0)	0 (0)	20 (100)
Directed to 7 suggestions for an active recovery	21 (100)	0 (0)	0 (0)	0 (0)	21 (100)
1 - Leave the house daily	14 (67)	7 (33)	0 (0)	0 (0)	14 (100)
2 - Make TV advert breaks active	21 (100)	0 (0)	0 (0)	0 (0)	21 (100)
3 - Stand up when waiting for something (e.g. bus, kettle)	7 (33)	14 (67)	0 (0)	0 (0)	7 (100)
4 - Tiptoe when waiting in a queue	0 (0)	21 (100)	N/A	N/A	N/A
5 - Increase your steps	18 (86)	3 (14)	0 (0)	0 (0)	18 (100)
6 - Sit to stand with no hands	2 (10)	19 (90)	0 (0)	0 (0)	2 (100)
7 - Treat the seat as a treat	7 (33)	14 (67)	0 (0)	2 (29)	5 (71)
Additional tailored top tip examples	17 (81)	4 (19)	0 (0)	0 (0)	17 (100)
**Total score**	**188 (68.9)**	**85 (31.1)**	**1 (0.5)**	**3 (1.6)**	**184 (97.9)**
**Smart Device (n=12)**					
LUMO and Smart Device communicate with each other	12 (100)	0 (0)	0 (0)	0 (0)	12 (100)
Patient shown how to lock and unlock the Smart Device	9 (75)	3 (15)	0 (0)	0 (0)	9 (100)
**Total score**	**21 (87.5)**	**3 (12.5)**	**0 (0)**	**0 (0)**	**21 (100)**
**LUMO App (n=12)**					
App provides you with information on sitting, standing and stepping	12 (100)	0 (0)	0 (0)	0 (0)	12 (100)
Patient shown where to find time spent sitting	12 (100)	0 (0)	0 (0)	0 (0)	12 (100)
Patient shown where to find how many times they have stood up	12 (100)	0 (0)	0 (0)	1 (8)	11 (92)
Patient shown where to find their step count	12 (100)	0 (0)	0 (0)	0 (0)	12 (100)
**Total score**	**48 (100)**	**0 (0)**	**0 (0)**	**1 (2.1)**	**47 (97.9)**
**Sit Time Panel (n=12)**					
Patient shown the pie chart for sitting, standing, stepping, lying down	12 (100)	0 (0)	0 (0)	0 (0)	12 (100)
Patient shown the hourly bar chart for all behaviours	12 (100)	0 (0)	0 (0)	1 (8)	11 (92)
Patient shown how to look back at previous days	10 (83)	2 (17)	0 (0)	5 (50)	5 (50)
Patient shown how to return to today's	5 (42)	7 (58)	0 (0)	0 (0)	5 (100)

information					
Patient shown how to return to the home screen	12 (100)	0 (0)	0 (0)	1 (8)	11 (92)
**Total score**	**51 (85.0)**	**9 (15.0)**	**0 (0)**	**7 (13.7)**	**44 (86.3)**
**Stand Ups Panel (n=12)**					
Patient shown the hourly bar chart for all behaviours	10 (83)	2 (17)	0 (0)	3 (30)	7 (70)
Patient shown how to look back at previous days	10 (83)	2 (17)	0 (0)	5 (50)	5 (50)
Patient shown how to return to today's information	7 (58)	5 (42)	0 (0)	2 (29)	5 (71)
Patient shown how to return to the home screen	9 (75)	3 (15)	0 (0)	0 (0)	9 (100)
**Total score**	**36 (75.0)**	**12 (25.0)**	**0 (0)**	**10 (27.8)**	**26 (72.2)**
**Steps Panel (n=12)**					
Patient shown the hourly bar chart for all behaviours	10 (83)	2 (17)	0 (0)	2 (20)	8 (80)
Patient shown how to look back at previous days	11 (92)	1 (8)	0 (0)	7 (64)	4 (36)
Patient shown how to return to today's information	7 (58)	5 (42)	0 (0)	4 (57)	3 (43)
Patient shown how to return to the home screen	11 (92)	1 (8)	0 (0)	1 (9)	10 (91)
**Total score**	**39 (81.3)**	**9 (18.8)**	**0 (0)**	**14 (35.9)**	**25 (64.1)**
**Vibration Prompt (n=12)**					
LUMO provides a vibration prompt when patient sits for 'too long'	12 (100)	0 (0)	0 (0)	1 (8)	11 (92)
Vibration will only go off once then the timer will reset	8 (67)	4 (33)	0 (0)	1 (12)	7 (88)
Patient chooses the duration of sitting before vibration occurs	12 (100)	0 (0)	0 (0)	2 (17)	10 (83)
**Total score**	**32 (88.9)**	**4 (11.1)**	**0 (0)**	**4 (12.5)**	**28 (87.5)**

Quality definitions for Education components: ‘Poor’, mentioned without examples; ‘Adequate’, explained without examples; ‘Excellent’, explained with examples.

Quality definitions for Feedback components were: ‘Poor’, mentioned without patient performing task; ‘Adequate’, explained without patient performing task; ‘Excellent’, explained with patients performing task.
