# Supplementary material for: Findings of the Chronic Obstructive Pulmonary Disease-Sitting and Exacerbations Trial (COPD-SEAT) in Reducing Sedentary Time Using Wearable and Mobile Technologies With Educational Support: Randomized Controlled Feasibility Trial
Source: JMIR Mhealth Uhealth. 2018 Apr 11;6(4):e84. doi: 10.2196/mhealth.9398 (PMC5917078; doi:10.2196/mhealth.9398)
Supplement: Multimedia Appendix 4 [file mhealth_v6i4e84_app4.pdf]

Supplementary File 3 Comparison of baseline versus follow-up responses to health questionnaires, stratified by study group, reported as mean (SD) unless otherwise stated.

	**Control (N=6)**		**Education (N=3)**		**Education+Feedback (N=8)**	
	**Baseline**	**Follow-up**	**Baseline**	**Follow-up**	**Baseline**	**Follow-up**
CAT score	24.5 (9.7)	23.8 (11.1)	31.0 (6.9)	22.7 (13.7)	22.0 (4.4)	21.6 (5.0)
Fatigue score [A]	32.6 (9.1)	34.0 (15.9)	12.0 (12.8)	19.0 (12.3)	25.9 (11.1)	25.9 (9.2)
No severe fatigue/severe fatigue	1/5	2/4	0/3	1/2	2/4	5/1
HADS depression score	10.0 (3.0)	8.3 (5.5)	12.0 (8.2)	8.3 (7.0)	3.6 (2.0)	4.4 (3.2)
Normal/borderline abnormally high/ abnormally high	1/2/3	3/0/3	1/0/2	1/1/1	8/0/0	6/2/0
HADS anxiety score	12.2 (5.5)	12.5 (6.0)	11.3 (8.0)	11.7 (8.0)	5.5 (2.1)	5.4 (3.5)
Normal/borderline abnormally high/ abnormally high	2/0/4	1/2/3	1/0/2	1/0/2	8/0/0	6/2/0
FESI score [B]	23.3 (10.5)	26.3 (13.3)	43.0 (20.7)	41.7 (20.8)	25.5 (9.5)	34.3 (3.3)

Abbreviations: CAT, COPD assessment test; FESI, falls efficacy scale international; HADS, hospital anxiety and depression scale

[A], Control N=5; Education+Feedback N=7; [B], Control N=4; Education+Feedback N=4
